# Supplementary material for: Modeling glioblastoma heterogeneity as a dynamic network of cell states
Source: Mol Syst Biol. 2021 Sep 16;17(9):e10105. doi: 10.15252/msb.202010105 (PMC8444284; doi:10.15252/msb.202010105)
Supplement: Supplementary file 6 — Source Data for Figure 5 [file MSB-17-e10105-s004.zip › Figure5A_sourcedata/GSEA_3071/hallmarks_stateI.GseaPreranked.1623742531165/HALLMARK_APOPTOSIS.html]

Details for gene set HALLMARK\_APOPTOSIS[GSEA]

|  || Dataset | state13071 |
| Phenotype | NoPhenotypeAvailable |
| Upregulated in class | na\_pos |
| GeneSet | HALLMARK\_APOPTOSIS |
| Enrichment Score (ES) | 0.37430498 |
| Normalized Enrichment Score (NES) | 2.005741 |
| Nominal p-value | 0.0 |
| FDR q-value | 0.0042277514 |
| FWER p-Value | 0.021 |
Table: GSEA Results Summary

  

Fig 1: Enrichment plot: HALLMARK\_APOPTOSIS      
 Profile of the Running ES Score & Positions of GeneSet Members on the Rank Ordered List

  

| PROBE | GENE SYMBOL | GENE\_TITLE | RANK IN GENE LIST | RANK METRIC SCORE | RUNNING ES | CORE ENRICHMENT || 1 | LUM |  |  | 4 | 2.203 | 0.0494 | Yes |
| 2 | DCN |  |  | 5 | 2.010 | 0.0956 | Yes |
| 3 | PDGFRB |  |  | 9 | 1.893 | 0.1381 | Yes |
| 4 | BGN |  |  | 24 | 1.568 | 0.1701 | Yes |
| 5 | TIMP1 |  |  | 47 | 1.381 | 0.1954 | Yes |
| 6 | LEF1 |  |  | 102 | 1.084 | 0.2046 | Yes |
| 7 | F2R |  |  | 105 | 1.067 | 0.2285 | Yes |
| 8 | SLC20A1 |  |  | 116 | 1.035 | 0.2494 | Yes |
| 9 | EMP1 |  |  | 122 | 1.012 | 0.2712 | Yes |
| 10 | LMNA |  |  | 247 | 0.739 | 0.2522 | Yes |
| 11 | GSN |  |  | 265 | 0.720 | 0.2638 | Yes |
| 12 | IFITM3 |  |  | 268 | 0.714 | 0.2796 | Yes |
| 13 | IL18 |  |  | 272 | 0.710 | 0.2950 | Yes |
| 14 | IRF1 |  |  | 276 | 0.705 | 0.3103 | Yes |
| 15 | MMP2 |  |  | 284 | 0.697 | 0.3243 | Yes |
| 16 | PDCD4 |  |  | 295 | 0.682 | 0.3371 | Yes |
| 17 | LGALS3 |  |  | 390 | 0.600 | 0.3236 | Yes |
| 18 | TNFSF10 |  |  | 395 | 0.590 | 0.3359 | Yes |
| 19 | IFNGR1 |  |  | 407 | 0.580 | 0.3461 | Yes |
| 20 | SQSTM1 |  |  | 412 | 0.578 | 0.3582 | Yes |
| 21 | RARA |  |  | 512 | 0.501 | 0.3409 | Yes |
| 22 | DNAJC3 |  |  | 533 | 0.489 | 0.3464 | Yes |
| 23 | NEDD9 |  |  | 538 | 0.487 | 0.3564 | Yes |
| 24 | CASP4 |  |  | 585 | 0.460 | 0.3536 | Yes |
| 25 | SMAD7 |  |  | 597 | 0.454 | 0.3609 | Yes |
| 26 | RHOB |  |  | 609 | 0.447 | 0.3679 | Yes |
| 27 | HMOX1 |  |  | 623 | 0.443 | 0.3743 | Yes |
| 28 | DDIT3 |  |  | 749 | 0.398 | 0.3472 | No |
| 29 | TIMP3 |  |  | 773 | 0.391 | 0.3495 | No |
| 30 | TSPO |  |  | 826 | 0.373 | 0.3430 | No |
| 31 | RNASEL |  |  | 947 | 0.340 | 0.3160 | No |
| 32 | CASP1 |  |  | 981 | 0.331 | 0.3140 | No |
| 33 | IGFBP6 |  |  | 1048 | 0.318 | 0.3021 | No |
| 34 | PPT1 |  |  | 1071 | 0.312 | 0.3029 | No |
| 35 | CAV1 |  |  | 1341 | 0.261 | 0.2308 | No |
| 36 | CD44 |  |  | 1430 | -0.251 | 0.2111 | No |
| 37 | CREBBP |  |  | 1518 | -0.259 | 0.1918 | No |
| 38 | DFFA |  |  | 1586 | -0.266 | 0.1784 | No |
| 39 | CASP3 |  |  | 1639 | -0.273 | 0.1696 | No |
| 40 | ENO2 |  |  | 1707 | -0.281 | 0.1566 | No |
| 41 | DNM1L |  |  | 1715 | -0.282 | 0.1611 | No |
| 42 | BCL2L1 |  |  | 1954 | -0.313 | 0.0992 | No |
| 43 | CDKN1A |  |  | 2083 | -0.332 | 0.0696 | No |
| 44 | BTG2 |  |  | 2318 | -0.375 | 0.0104 | No |
| 45 | DPYD |  |  | 2336 | -0.382 | 0.0142 | No |
| 46 | BID |  |  | 2405 | -0.393 | 0.0035 | No |
| 47 | WEE1 |  |  | 2426 | -0.397 | 0.0068 | No |
| 48 | PLAT |  |  | 2501 | -0.415 | -0.0051 | No |
| 49 | VDAC2 |  |  | 2535 | -0.424 | -0.0050 | No |
| 50 | BMP2 |  |  | 2589 | -0.436 | -0.0104 | No |
| 51 | BAX |  |  | 2660 | -0.457 | -0.0202 | No |
| 52 | APP |  |  | 2770 | -0.495 | -0.0404 | No |
| 53 | TOP2A |  |  | 2842 | -0.517 | -0.0492 | No |
| 54 | CDK2 |  |  | 2863 | -0.526 | -0.0429 | No |
| 55 | SATB1 |  |  | 2870 | -0.527 | -0.0325 | No |
| 56 | ERBB3 |  |  | 3038 | -0.625 | -0.0666 | No |
| 57 | CDKN1B |  |  | 3065 | -0.646 | -0.0594 | No |
| 58 | CCND1 |  |  | 3072 | -0.655 | -0.0461 | No |
| 59 | GPX4 |  |  | 3103 | -0.679 | -0.0392 | No |
| 60 | BTG3 |  |  | 3148 | -0.727 | -0.0352 | No |
| 61 | BRCA1 |  |  | 3185 | -0.765 | -0.0281 | No |
| 62 | TGFB2 |  |  | 3251 | -0.852 | -0.0274 | No |
| 63 | FEZ1 |  |  | 3297 | -0.928 | -0.0192 | No |
| 64 | CTH |  |  | 3336 | -0.990 | -0.0075 | No |
| 65 | HMGB2 |  |  | 3390 | -1.184 | 0.0043 | No |
| 66 | CLU |  |  | 3424 | -1.329 | 0.0252 | No |
Table: GSEA details [plain text format]

  

Fig 2: HALLMARK\_APOPTOSIS: Random ES distribution      
 Gene set null distribution of ES for **HALLMARK\_APOPTOSIS**
